# Einfluss einer tiefen Implantation auf Reizleitungsstörungen nach Transkatheter-Aortenklappenimplantation

**DOI:** 10.1007/s00399-021-00784-1

**Published:** 2021-07-14

**Authors:** Mohammed Saad, Yannic Klaus, Paul Buhse, Thomas Puehler, Georg Lutter, Hatim Seoudy, Derk Frank

**Affiliations:** 1 Department of Internal Medicine III, Cardiology and Angiology, University Hospital Schleswig-Holstein, Campus Kiel, Kiel, Deutschland; 2 DZHK (German Centre for Cardiovascular Research), partner site Hamburg/Kiel/Lübeck, Kiel, Deutschland; 3 Department of Cardiac and Vascular Surgery, University Hospital Schleswig-Holstein, Campus Kiel, Kiel, Deutschland

**Keywords:** TAVI, Implantationstiefe, Schrittmacher, Überleben, Linksschenkelblock, TAVI, Implantation depth, Pacemaker, Survival, Left bundle branch block

## Abstract

**Hintergrund:**

Reizleitungsstörungen sind häufige Komplikationen der Transkatheter-Aortenklappenimplantation (TAVI). Ein Einflussfaktor ist die bisher nicht standardisiert bestimmte Implantationstiefe.

**Fragestellung:**

Gibt es Unterschiede zwischen den anatomischen Regionen bei tiefer Implantation hinsichtlich neuer Reizleitungsstörungen und Überleben?

**Material und Methoden:**

Retrospektive Kohortenanalyse, 420 Patienten mit transfemoraler TAVI mit Klappenprothesen der neuen Generation, davon 352 Patienten ohne vorbestehenden Schrittmacher für Analysen zur neuen Schrittmacherimplantation. Auswertung der fluoroskopisch gemessenen Implantationstiefen an der non- (NCC) und der links-koronaren Tasche (LCC) sowie der aus Patientenakten ersichtlichen Verläufe. Die tiefe Implantation definierte sich als tiefes Quartil der Implantationstiefe der jeweiligen Prothese. Das Überleben ergab sich aus einem 2‑jährigen Follow-up.

**Ergebnisse und Diskussion:**

Die tiefe Implantation war nur am NCC mit erhöhter Schrittmacherimplantationsrate assoziiert (*p* = 0,013), am LCC mit dem häufigeren Auftreten eines permanenten Linksschenkelblocks (*p* = 0,014). Neue oder vorbestehende Schrittmacherimplantationen hatten keinen Einfluss auf das 2‑jährige Überleben. Eine tiefe Implantation war nicht mit einer schlechteren Überlebensprognose assoziiert. Die Implantationstiefe könnte bezüglich der Bedeutung für neue Schrittmacherimplantationen standardisiert am NCC gemessen werden. In der Eingriffsplanung könnte eine Abschätzung der Implantationstiefe am NCC oder LCC relevant für den Verlauf nach TAVI sein. Die Schrittmacherimplantation kann wie auch die tiefe Implantation am LCC oder NCC, bei Abwesenheit von mittel- bis hochgradigen paravalvulären Leckagen, als prognostisch sicher gewertet werden.

## Abstract (en)

**Background:**

Conduction disturbances are common complications of transcatheter aortic valve implantation (TAVI). One influencing factor is implantation depth (ID) of the TAVI prosthesis. Since this should be standardized, a differentiated consideration of ID is necessary.

**Objective:**

Examination of the impact of ID at different anatomical regions of the left ventricular outflow tract on new conduction disturbances, new permanent pacemaker implantation (PPI) and survival.

**Materials and methods:**

The retrospective cohort study included 420 patients who underwent transfemoral TAVI with new-generation devices, including 352 patients without pre-existing pacemakers, for analyses on new pacemaker implantation. Of them, 46 patients underwent PPI. ID at non- (NCC) and left-coronary cusp (LCC) were measured using fluoroscopy after valve implantation. Deep ID was defined as the 4th quartile of each prosthesis’ ID. Survival was determined from the two-year follow-up.

**Results and conclusions:**

Deep ID was associated with higher PPI rate only at NCC (*p* = 0.013). At LCC deep ID resulted in more frequent permanent left bundle branch block (*p* = 0.014). DI, preexisting pacemaker or new PPI did not affect 2‑year survival. No patients with new PPI suffered moderate to severe paravalvular leak. ID at NCC could be a predictor for PPI after TAVI. A detailed consideration of ID in intervention planning might predict outcome after TAVI and common complications. PPI after TAVI and deep ID at NCC and LCC can be considered safe regarding survival in absence of moderate-to-severe paravalvular leak.

## Hintergrund und Fragestellung

Die Bedeutung der Transkatheter-Aortenklappenimplantation (TAVI) als Therapieoption der hochgradigen Aortenklappenstenose hat in den vergangenen Jahren zugenommen [1]. Die Implantationstiefe (IT) im linksventrikulären Ausflusstrakt (LVOT) ist als Einflussfaktor für neue Schrittmacherimplantationen und den neuen Linksschenkelblock als wichtige Komplikationen beschrieben [12]. Eine möglichst hohe Implantation mit geringem Prothesenanteil im LVOT gilt als protektiv [9, 12]. Eine differenzierte Betrachtung dieses Risikofaktors ist nicht hinreichend beschrieben. Da die einzelnen Taschen der Aortenklappe verschiedene anatomische Beziehungen zum kardialen Reizleitungssystem haben, ist eine besondere Relevanz von tiefen Implantationen an verschiedenen Lokalisationen im LVOT denkbar und wäre hilfreich für die Etablierung einer standardisierten Messmethode.

## Studiendesign und Untersuchungsmethoden

In dieser retrospektiven Kohortenstudie wurden Patienten eingeschlossen, die sich zwischen Februar 2014 und Dezember 2017 einer transfemoralen TAVI mit einer Prothese der neuen Generation am Universitätsklinikum Schleswig-Holstein, Campus Kiel, unterzogen haben. Patienten, die keinen vorbestehenden Schrittmacher hatten, konnten in der Analyse der neuen Herzschrittmacherimplantationen nach TAVI berücksichtigt werden. Die implantierten Prothesen waren die ballonexpandierbare „Edwards SAPIEN 3™“ (Edwards Lifesciences Corporation, Irvine, Kalifornien, Vereinigte Staaten) und die selbstexpandierende „Medtronic CoreValve™ Evolut R™“ (Medtronic Inc, Minneapolis, Minnesota, Vereinigte Staaten). Ausgeschlossen wurden Patienten mit Prothesen älterer Generationen, anderen Prothesen oder transapikalem und transaortalem Zugang. In Anlehnung an die VARC-3-Kriterien [10] wurden die Aspekte „Wechsel zu offenchirurgischem Verfahren“, „lebensbedrohliche Blutung“ und „Schlaganfall“ nach ihrer dortigen Definition registriert.

## Messung der IT

Grundlage der IT-Messung war die Auswertung der periprozeduralen Röntgenaufnahmen. Diese konnten mittels der Software „Xcelera“ (Version R4.1L1-SP1, Philips Medical Systems Nederland B.V., Niederlande, 2014) hinsichtlich der absoluten IT unterhalb des nativen Aortenanulus vermessen werden. Es waren der Prothesenanteil an der non-koronaren Tasche (NCC) sowie an der links-koronaren Tasche (LCC) einsehbar. An beiden Lokalisationen im LVOT wurde die IT separat durch die Länge des Prothesenrahmens vom nativen Anulus bis zum intraventrikulären Ende des Rahmens gemessen. Die Eichung des Programms erfolgte durch Autokalibrierung. Gemessen wurde mittels eines integrierten Messtools.

Das Quartil Q4 umfasste für Patienten mit einer ballonexpandierbaren Prothese eine IT von ≥ 8 mm am NCC und ≥ 7 mm am LCC. Für Patienten mit einer selbstexpandierenden Prothese definierte sich Q4 als IT von ≥ 9 mm am NCC und am LCC von ≥ 10 mm im LVOT.

## EKG, Herzschrittmacher und Echokardiographie

Das zeitnächste verfügbare EKG, höchstens 60 Tage vor dem Eingriff sowie postinterventionell bis 30 Tage nach TAVI, wurde ausgewertet. Im Fall von fehlenden EKGs wurde auf ärztlich dokumentierte EKG-Befunde in der Patientenakte und in Arztbriefen zurückgegriffen. Ein AV-Block I° bestand ab einer PQ-Zeit von ≥ 200 ms, eine verbreiterte QRS-Dauer bei ≥ 120 ms. Ein permanenter Linksschenkelblock musste zur Wertung bis zum letzten verfügbaren EKG 30 Tage nach TAVI bestehen.

Eine neue Schrittmacherimplantation innerhalb von 30 Tagen nach TAVI betraf neue 1‑, 2‑ oder 3‑Kammer-Schrittmachersysteme. Ein Teil der Patienten hatte bereits vor TAVI einen vorbestehenden Schrittmacher. Befunde aus der Patientenakte oder aus Arztbriefen lieferten präinterventionelle echokardiographische Daten. Ein *Oversizing *wurde als Verhältnis von Prothesengröße zu echokardiographisch bestimmtem Anulusdiameter sowie LVOT-Diameter errechnet.

## Statistische Analyse

Statistische Auswertungen und Grafikerstellungen erfolgten mit IBM SPSS Statistics, Version 25, Microsoft Excel 2016 und GraphPad Prism, Version 8.4.3. Das tiefe Quartil der IT der jeweiligen Prothese definierte die tiefe Implantation. Dies wurde jeweils für die IT am NCC und am LCC bestimmt. Kategoriale Variablen wurden als relative und absolute Häufigkeiten gewerteter Fälle dargestellt, stetige Variablen als Median mit Interquartilsabstand. Mit Hilfe des Chi-Quadrat-Tests, Fisher′s Exakt-Tests oder Mann-Whitney-U-Tests wurde die statistische Signifikanz von Unterschieden zwischen den verglichenen Gruppen bestimmt. Es wurde zweiseitig getestet. Überlebenszeitanalysen erfolgten mittels Kaplan-Meier-Verfahren und Log-rank-Test. Ein *p*-Wert < 0,05 galt als statistisch signifikant.

## Ergebnisse

Von 420 Patienten wurden 250 (59,5 %) mit einer Edwards SAPIEN 3™ und 170 (40,5 %) mit einer Medtronic CoreValve™ Evolut R™ versorgt. 352 (83,8 %) hatten keinen vorbestehenden Schrittmacher. Davon erhielten 46 (13,1 %) nach TAVI einen neuen Herzschrittmacher. Bei 39 (84,8 %) Patienten war ein AV-Block III° die häufigste Indikation für die Schrittmacherimplantation. Im Anhang in Tab. 3 sind die implantierten Schrittmacher mit ihren Indikationen aufgeführt. Tab. 1 zeigt die allgemeinen Basischarakteristika dieser Patienten und den Vergleich der Patienten mit und ohne Schrittmacherimplantation nach TAVI. Dabei wiesen Patienten mit neuer Schrittmacherimplantation häufiger zerebrovaskuläre Vorerkrankungen auf.

**Table 1:** 

Gesamt		Neue Schrittmacherimplantation		*p*-Wert
*n* = 352		Nein *n* = 306	Ja *n* = 46	
**Alter**	81 (78–85)	81 (78–86)	81 (77,75–84,25)	0,416
**Männliches Geschlecht**	40,9 % (144/352)	41,5 % (127/306)	37 % (17/46)	0,631
**Body-Mass-Index (kg/m**^**2**^**)**	26,51 (23,83–29,75)	26,48 (23,74–29,68)	26,83 (24,55–30,43)	0,280
**STS-Score (%)**	3,94 (2,55–6,09)	3,95 (2,54–6,3)	3,91 (2,6–5,21)	0,779
**Logistischer EuroSCORE (%)**	14,2 (8,97–23,08)	14,4 (8,97–23,47)	12,69 (8,73–18,28)	0,359
**EuroSCORE II (%)**	3,94 (2,49–6,4)	3,99 (2,44–6,42)	3,87 (2,69–6,15)	0,884
**NYHA-Stadium**				
III	61 % (214/351)	61 % (186/305)	60,9 % (28/46)	1,000
IV	11,7 % (41/351)	10,8 % (33/305)	17,4 % (8/46)	0,217
**Koronare Herzkrankheit**	67,6 % (238/352)	69 % (211/306)	58,7 % (27/46)	0,179
**Herzchirurgische Voroperation**	15,7 % (55/351)	15,4 % (47/305)	17,4 % (8/46)	0,669
**Vorhofflimmern/-flattern**	37,9 % (133/351)	38,7 % (118/305)	32,6 % (15/46)	0,515
**pAVK**	9,1 % (32/350)	9,9 % (30/304)	4,3 % (2/46)	0,284
**Zerebrovaskuläre Erkrankung**	20,3 % (71/350)	18,4 % (56/304)	32,6 % (15/46)	*0,031*
**Arterielle Hypertonie**	91,5 % (322/352)	91,2 % (279/306)	93,5 % (43/46)	0,781
**Dyslipidämie**	48,4 % (170/351)	48,9 % (149/305)	45,7 % (21/46)	0,753
**Diabetes mellitus**	29,3 % (103/351)	29,5 % (90/305)	28,3 % (13/46)	1,000
**Niereninsuffizienz (GFR** **<** **30** **ml/min)**	12,8 % (45/352)	12,1 % (37/306)	17,4 % (8/46)	0,342
**COPD**	10,9 % (38/350)	9,9 % (30/304)	17,4 % (8/46)	0,131

Alle stetigen Variablen sind als Median und Interquartilsabstand dargestellt, alle kategoriellen in Prozent. Prozentwerte können aufgrund von Rundungen nicht immer 100 ergeben. Kursive Werte zeigen statistisch signifikante Unterschiede zwischen den Gruppen an

*STS* The Society of Thoracic Surgeons-Score *NYHA* New York Heart Association, *pAVK* periphere arterielle Verschlusskrankheit,* GFR* glomeruläre Filtrationsrate,* COPD* chronisch-obstruktive Lungenerkrankung

Basischarakteristika aus dem EKG vor TAVI sowie der Echokardiographie sind im Anhang in Tab. 4 und 5 aufgeführt. Ein vorbestehender Rechtsschenkelblock war mit neuer Schrittmacherimplantation assoziiert, ebenso eine vorbestehende Verbreiterung des QRS-Komplexes auf über 120 ms.

Unter schrittmacherimplantierten Patienten fand sich häufiger eine neue Verbreiterung des QRS-Komplexes auf über 120 ms nach TAVI und im postinterventionellen EKG häufiger ein neuer Rechtsschenkelblock. In Tab. 2 sind prozedurale Charakteristika im Vergleich zwischen Patienten mit und ohne postinterventionelle Schrittmacherimplantation dargestellt.

**Table 2:** 

Gesamt		Neue Schrittmacherimplantation		*p*-Wert
*n* = 352		Nein *n* = 306	Ja *n* = 46	
**Prozedurale Charakteristika**				
Klappentyp				0,200
SAPIEN 3™	59,1 % (208/352)	60,5 % (185/306)	50 % (23/46)	–
CoreValve™ Evolut R™	40,9 % (144/352)	39,5 % (121/306)	50 % (23/46)	–
**Prothesengröße (mm)**				0,588
23	22,2 % (78/352)	22,2 % (68/306)	21,7 % (10/46)	–
26	37,8 % (133/352)	38,9 % (119/306)	30,4 % (14/46)	–
29	34,9 % (123/352)	33,7 % (103/306)	43,5 % (20/46)	–
34	5,1 % (18/352)	5,2 % (16/306)	4,3 % (2/46)	–
Prothesengröße/Anulusdiameter	1,16 (1,083–1,261)	1,15 (1,083–1,261)	1,209 (1,04–1,318)	0,711
Prothesengröße/LVOT-Diameter	1,278 (1,182–1,381)	1,278 (1,182–1,381)	1,318 (1,171–1,399)	0,627
Nachdehnen	38,2 % (129/338)	38 % (112/295)	39,5 % (17/43)	0,868
VARC: Wechsel zu offenchirurgischem Verfahren	0,3 % (1/352)	0,3 % (1/306)	0 % (0/46)	1,000
VARC: Lebensbedrohliche Blutung	2,6 % (9/352)	2,3 % (7/306)	4,3 % (2/46)	0,333
VARC: Schlaganfall	0,6 % (2/352)	0,7 % (2/306)	0 % (0/46)	1,000
Paravalvuläres Leck (moderat/hochgradig)	2,9 % (10/341)	3,4 % (10/298)	0 % (0/43)	0,621
**Elektrokardiographische Daten**				
Neuer permanenter Linksschenkelblock	19,4 % (67/346)	19,9 % (60/302)	15,9 % (7/44)	0,684
Neuer Rechtsschenkelblock	2,1 % (7/340)	1 % (3/300)	10 % (4/40)	*0,004*
Neuer linksanteriorer Hemiblock	1,8 % (6/329)	1,7 % (5/299)	3,3 % (1/30)	0,439
Neuer linksposteriorer Hemiblock	0 % (0/325)	0 % (0/298)	0 % (0/27)	–
Neue QRS-Dauer ≥ 120 ms	23,3 % (81/347)	20,2 % (61/302)	44,4 % (20/45)	*0,001*
Neuer AV-Block I° (wenn Sinusrhythmus)	15,2 % (39/256)	14 % (33/236)	30 % (6/20)	0,095
Neuer AV-Block III°	16,7 % (58/348)	5,6 % (17/302)	89,1 % (41/46)	*<0,001*
**Implantationstiefen**				
Implantationstiefe am NCC (mm)				*0,013*
Q1‑3	77,8 % (274/352)	80,1 % (245/306)	63 % (29/46)	–
Q4	22,2 % (78/352)	19,9 % (61/306)	37 % (17/46)	–
Implantationstiefe am LCC (mm)				1,000
Q1‑3	81,5 % (287/352)	81,4 % (249/306)	82,6 % (38/46)	–
Q4	18,5 % (65/352)	18,6 % (57/306)	17,4 % (8/46)	–

Alle stetigen Variablen sind als Median und Interquartilsabstand dargestellt, alle kategoriellen in Prozent. Prozentwerte können aufgrund von Rundungen nicht immer 100 ergeben. Kursive Werte zeigen statistisch signifikante Unterschiede zwischen den Gruppen an

*LVOT* linksventrikulärer Ausflusstrakt, *VARC* Valve Academic Research Consortium, *AV-Block* atrioventrikulärer Block, *NCC* non-koronare Tasche, *LCC* links-koronare Tasche, *Q* Quartil

**Figure 1:**
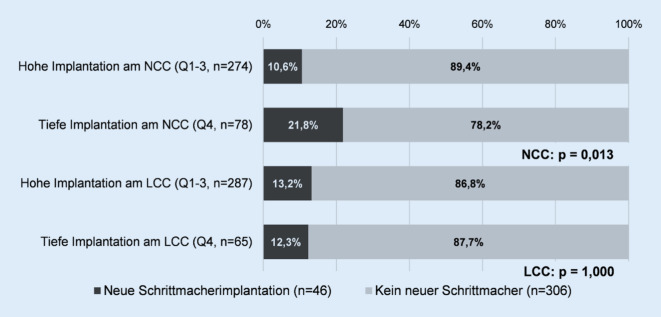

Wie Tab. 2 zeigt, fand sich eine tiefe Implantation am NCC signifikant häufiger unter Patienten mit neuer Schrittmacherimplantation nach TAVI (19,9 % vs. 37 %; *p* = 0,013). Am LCC dagegen gab es keinen Unterschied bezüglich der Häufigkeit tiefer Implantationen bei Patienten ohne oder mit Schrittmacherimplantation nach TAVI. Die relativen Häufigkeiten der neuen Schrittmacherimplantationen bei hoher und tiefer Implantation am NCC und LCC sind in Abb. 1 dargestellt.

**Figure 2:**
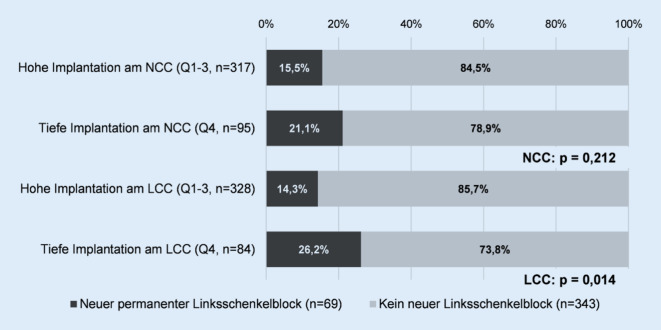

Eine tiefe Implantation am LCC zeigte sich als relevant beim Auftreten eines neuen permanenten Linksschenkelblocks nach TAVI. Wie in Abb. 2 illustriert, war der Anteil von Patienten mit permanentem Linksschenkelblock nach TAVI signifikant größer bei tiefer Implantation am LCC (14,3 % vs. 26,2 %; *p* = 0,014). Bei tiefer Implantation am NCC konnte dieser Unterschied nicht beobachtet werden.

Mit einer tiefen Implantation war ein großes *Oversizing*, insbesondere am LVOT (*n* = 414, NCC: Prothesengröße/LVOT-Diameter 1,278 [1,182–1,381] vs. 1,318 [1,208–1,45]; *p* = 0,019; LCC: Prothesengröße/LVOT-Diameter 1,261 [1,182–1,381] vs. 1,368 [1,261–1,45], *p* < 0,001) statistisch assoziiert. Große Prothesen wurden sowohl am NCC als auch am LCC häufiger tief implantiert.

## Überleben

**Figure 3:**
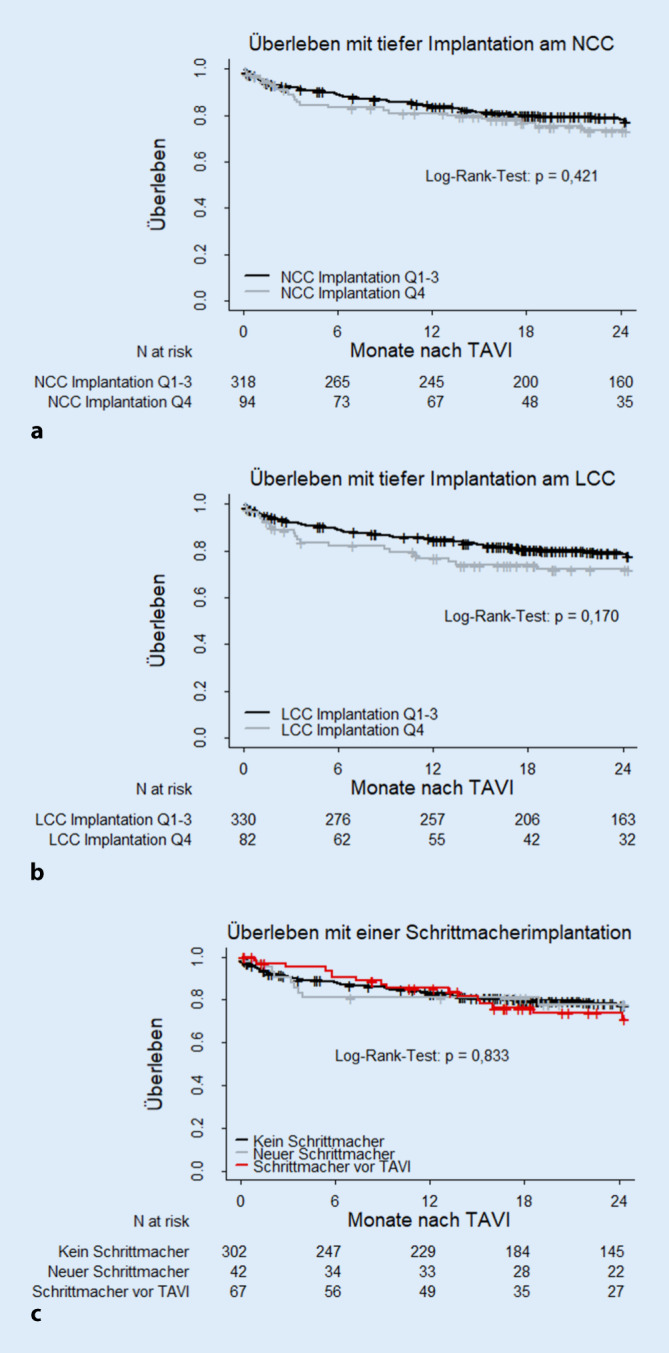

Beim 2‑jährigen Follow-up zeigte sich zwischen 411 Patienten mit und ohne neue Schrittmacherimplantation sowie vorbestehendem Herzschrittmacher kein signifikanter Unterschied in der Überlebensprognose. Es konnten jeweils 412 Patienten hinsichtlich des Überlebens nach TAVI mit einer tiefen Implantation am NCC und am LCC ausgewertet werden. Die tiefe Implantation am NCC hatte wie die tiefe LCC-seitige Implantation keinen signifikanten Einfluss auf das Gesamtüberleben. Die Kaplan-Meier-Kurven zur Darstellung der Überlebenszeitanalysen sind in Abb. 3 dargestellt.

## Diskussion

### Neue Herzschrittmacherimplantation

Die Ergebnisse der vorliegenden Studie unterstreichen die Wichtigkeit der differenzierten Betrachtung des Einflussfaktors IT bei Abwesenheit von mittel- bis hochgradigen paravalvulären Leckagen. Die tiefe Implantation im LVOT erwies sich nur am NCC als signifikant mit neuen Schrittmacherimplantationen assoziiert. Dagegen gab es zwischen tief implantierten Schrittmachern am LCC und höher implantierten keinen Unterschied in der Schrittmacherrate. Die tiefe Implantation am LCC stand dagegen signifikant mit dem Auftreten eines neuen permanenten Linksschenkelblocks in Verbindung. Dieser Trend ließ sich bei tiefer NCC-seitiger Implantation ebenfalls beobachten, verfehlte jedoch die statistische Signifikanz. Diese Erkenntnisse lassen vermuten, dass bei schiefer Implantation im LVOT einer Prothese der neuen Generation abhängig von der Lokalisation des tief implantierten Prothesenanteils unterschiedliche Komplikationen absehbar wären. Die anatomische Nähe des kardialen Reizleitungssystems zum NCC besitzt bereits, z. B. bei Katheterablationen, eine therapeutische Bedeutung [14]. Die Bedeutung der IT am NCC für neue Schrittmacherimplantationen lässt sich durch die anatomischen Lagebeziehungen des Reizleitungssystems zum NCC gut begründen. Eine weitere Studie konnte den Zusammenhang von neuer Schrittmacherimplantation mit einem hohen Kalzifizierungsgrad in der anatomischen Zielzone der Prothesenimplantation nur am NCC, jedoch nicht an LCC oder der rechts-koronaren Tasche (RCC) herstellen [11]. Eine asymmetrische Verteilung von Kalzifizierungen steht als mögliche Ursache von schrägen Implantationen zur Diskussion und könnte zu ungleichmäßiger mechanischer Manipulation am Reizleitungssystem führen [18]. Gonska et al. [7] konnten ebenfalls einen Zusammenhang des Kalzifizierungsgrades am NCC, nicht jedoch am LCC beobachten. Die IT könnte von den anatomischen Bedingungen ebenso wie vom Kalzifizierungsmuster abhängen. Als Erkrankungen, die mit einer Kalzifizierung im Sinne von Arteriosklerose einhergehen, hatten schrittmacherimplantierte Patienten häufiger eine zerebrovaskuläre Vorerkrankung. Ein höherer Verkalkungsgrad könnte auch für die native Aortenklappe denkbar sein.

Prädiktor für Schrittmacherimplantationen nach TAVI war übereinstimmend mit bestehenden Studien ein vorbestehender Rechtsschenkelblock im EKG [6, 7, 12, 16]. In der vorliegenden Studie war auch ein neuer Rechtsschenkelblock nach TAVI ein Prädiktor für die anschließende Schrittmacherimplantation. Die vorbestehende QRS-Dauer von mindestens 120 ms war analog ein Risikofaktor, was sich mit bestehenden Studien deckt [9, 16, 17]. Auch die QRS-Verbreiterung nach TAVI auf über 120 ms führte häufiger zu Schrittmacherimplantationen, während ein neuer Linksschenkelblock dies im Gegensatz zum neuen Rechtsschenkelblock nicht tat.

Eine Unterteilung des Kollektivs nach Prothese ergab hier keinen signifikanten Unterschied bezüglich der Rate neuer Schrittmacherimplantationen. Unter Prothesen älterer Generationen erwies sich eine ballonexpandierbare Prothese gegenüber einer selbstexpandierenden als überlegen im Hinblick auf die Vermeidung einer neuen Schrittmacherimplantation [20]. Dieser Unterschied verliert jedoch bei Betrachtung der neuen Generation von TAVI-Prothesen mehr an Bedeutung [20]. Beim isolierten Vergleich von Prothesen der neuen Generation konnte im Einklang mit dieser Studie die Schrittmacherrate nach TAVI bereits als ähnlich beobachtet werden [5]. Unterschiede zwischen verschiedenen Prothesentypen scheinen bezüglich neuer Schrittmacherimplantationen im Zuge technischer Weiterentwicklungen weniger relevant.

### Überleben

Bestehende Studien kamen zum gleichen Ergebnis, indem sie keinen Zusammenhang der neuen Schrittmacherimplantation mit der Überlebensprognose beobachten konnten [2, 4]. Auch große Studien wie von Regueiro et al. bestätigen diese Annahme [15]. Patienten mit vorbestehenden Schrittmachern wurden dabei oft ausgeschlossen, und es wurden Prothesen älterer Generationen verwendet. Jegliche Schrittmacherimplantationen waren demnach keine prognoselimitierende Maßnahme.

Die vorliegenden Ergebnisse legen die prognostische Sicherheit einer tiefen Implantation an NCC und LCC ohne mittel- bis hochgradige paravalvuläre Leckagen nahe. Vavuranakis et al. [19] verwendeten die mittlere IT bei vergleichbarer Messmethode und definierten 4 mm als Grenzwert zwischen hoher und tiefer Implantation von selbstexpandierenden Prothesen. Nach einjährigem Follow-up konnten die Autoren keinen Überlebensunterschied detektieren. Der Fokus dieser Studie lag auf einer tiefen statt einer besonders hohen Implantation. Die Ergebnisse widersprachen sich jedoch nicht. Das Überleben bei tiefer Implantation am LCC war tendenziell schlechter. Dies könnte mutmaßlich an der höheren Rate neuer permanenter Linksschenkelblockierungen liegen, die mit tiefer LCC-seitiger Implantation einhergingen und in manchen Studien als potenziell prognoselimitierend diskutiert wurden [8, 13, 15].

## Limitationen

In dieser Studie wurden zwei verschiedene Prothesen der neuen Generation untersucht. Die Wahl der Prothese kann damit ein möglicher nicht berücksichtigter Störfaktor sein. Durch Messung der IT in ganzen Millimetern war eine exakte Aufteilung in Quartile nicht möglich, sodass die Aufteilung als Näherung zu verstehen ist und potenziell Ergebnisse verzerren kann. Ein Kalziumscoring zum Vergleich mit anderen Arbeiten [7, 11, 18] fand nicht statt. Ebenso erfolgte keine Reevaluation der Schrittmacherindikationen, die möglicherweise die tatsächliche Schrittmacherrate verändert hätte [3]. Im Rahmen der retrospektiven Analyse waren rekonstruierbare Datensätze oft lückenhaft, sodass die gewerteten Fallzahlen variierten.

## Fazit für die Praxis

- Die Wahl kleinerer Prothesengrößen, geringeres *Oversizing* oder eine neue Implantationstechnik (z. B. Implantation in „cusp overlap view“) vermag tiefe Implantationen zu vermeiden.
- Sollte eine tiefe Implantation am NCC unausweichlich sein, ist eine Schrittmacherimplantation nach TAVI wahrscheinlicher.
- Bei unumgänglicher tiefer Implantation am LCC ist ein neuer Linksschenkelblock mit höherer Wahrscheinlichkeit permanent.
- Eine Empfehlung zur einheitlichen Messung der IT als Risikofaktor für postprozedurale Schrittmacherimplantationen könnte sich auf die IT am NCC fokussieren.
- Patienten mit tiefer NCC-seitiger Implantation bedürfen strengerer Überwachung auf Störungen des kardialen Reizleitungssystems.
- Eine tiefe Implantation am NCC oder LCC beeinflusst die Überlebensprognose nicht.
- Die Überlebensprognose unterliegt nicht dem Einfluss einer neuen oder vorbestehenden Schrittmacherimplantation.

## Anhang

**Table 3:**

–	1‑Kammer-Schrittmacher	2‑Kammer-Schrittmacher	3‑Kammer-Schrittmacher	Total
AV-Block III°	66,7 % (2/3)	86,7 % (26/30)	84,6 % (11/13)	84,8 % (39/46)
SA-Block	0 (0 %)	6,7 % (2/30)	0 (0 %)	4,4 % (2/46)
Symptomatische Bradykardie	0 (0 %)	3,3 % (1/30)	7,7 % (1/13)	4,4 % (2/46)
Bradyarrhythmia absoluta	0 (0 %)	3,3 % (1/30)	7,7 % (1/13)	4,4 % (2/46)
Brady-Tachy-Syndrom	33,3 % (1/3)	0 (0 %)	0 (0 %)	2,2 % (1/46)
Total	6,5 % (3/46)	65,2 % (30/46)	28,3 % (13/46)	100 % (46/46)

*AV-Block* Atrioventrikulärer Block, *SA-Block* Sinuatrialer Block

**Table 4:**

Total		Neue Schrittmacherimplantation		*p*-Wert
*n* = 352		Nein *n* = 306	Ja *n* = 46	
Sinusrhythmus	78,2 % (273/349)	78,2 % (237/303)	78,3 % (36/46)	1,000
Vorhofflimmern	21,8 % (76/349)	21,8 % (66/303)	21,7 % (10/46)	1,000
AV-Block I° (wenn Sinusrhythmus)	21,1 % (57/270)	22,6 % (53/235)	11,4 % (4/35)	0,182
Rechtsschenkelblock	9,2 % (32/347)	6,6 % (20/302)	26,7 % (12/45)	*<* *0,001*
Linksschenkelblock	7,5 % (26/347)	7,6 % (23/302)	6,7 % (3/45)	1,000
Linksanteriorer Hemiblock	11,2 % (39/347)	10,3 % (31/302)	17,8 % (8/45)	0,136
Linksposteriorer Hemiblock	0 % (0/364)	0 % (0/302)	0 % (0/45)	–
QRS-Dauer ≥ 120 ms	19,8 % (69/348)	17,5 % (53/302)	34,8 % (16/46)	*0,010*

Alle stetigen Variablen sind als Median und Interquartilsabstand dargestellt, alle kategoriellen als %. Prozentwerte können aufgrund von Rundungen nicht immer 100 ergeben. Kursive Werte zeigen statistisch signifikante Unterschiede zwischen den Gruppen an

*AV-Block* Atrioventrikulärer Block

**Table 5:**

Total		Neue Schrittmacherimplantation		*p*-Wert
*n* = 352		Nein *n* = 306	Ja *n* = 46	
Klappenöffnungsfläche (cm^2^)	0,7 (0,6–0,9)	0,7 (0,6–0,9)	0,7 (0,6–0,8)	0,398
Mittlerer transvalvulärer Druckgradient (mm Hg)	40 (31–51)	40 (31–52)	43 (33–50)	0,827
Aortenanulus (mm)	22,5 (21–24)	22,5 (21–24)	23 (21–24)	0,935
LVOT Diameter (mm)	21 (19–22)	21 (19–22)	21 (19–22)	0,726
LAes Diameter (mm)	45 (40–50)	45 (40–50)	44 (40,25–48)	0,554
IVSd (mm)	14 (13–15)	14 (13–16)	14 (13–15)	0,760
LVEDD (mm)	46 (41–51)	46 (40–51)	47,5 (42–53)	0,236
LVPWd (mm)	13 (11–15)	13 (11–14)	13 (11–15)	0,893
LVEF < 55 %	35,2 % (124/352)	35,6 % (109/306)	32,6 % (15/46)	0,743
Aorteninsuffizienz (moderat/hochgradig)	10,4 % (35/338)	11,1 % (33/296)	4,8 % (2/42)	0,282
Mitralinsuffizienz (moderat/hochgradig)	18,9 % (64/339)	19,5 % (57/293)	15,2 % (7/46)	0,685
Trikuspidalinsuffizienz (moderat/hochgradig)	10 % (30/301)	9,9 % (26/263)	10,5 % (4/38)	0,779

Alle stetigen Variablen sind als Median und Interquartilsabstand dargestellt, alle kategoriellen als %. Prozentwerte können aufgrund von Rundungen nicht immer 100 ergeben. Kursive Werte zeigen statistisch signifikante Unterschiede zwischen den Gruppen an

*LVOT* Linksventrikulärer Ausflusstrakt, *LAes* Linkes Atrium endsystolisch, *IVSd* Diastolische Septumdicke *LVEDD* Linksventrikulärer enddiastolischer Durchmesser,* LVPWd* Diastolische Hinterwanddicke,* LVEF* Linksventrikuläre Ejektionsfraktion
